# Reducing *Mcl-1* gene dosage induces dopaminergic neuronal loss and motor impairments in *Park2* knockout mice

**DOI:** 10.1038/s42003-019-0366-x

**Published:** 2019-04-04

**Authors:** Susanna Ekholm-Reed, Robert Baker, Alexandre R. Campos, David Stouffer, Martha Henze, Dieter A. Wolf, Jeanne F. Loring, Elizabeth A. Thomas, Steven I. Reed

**Affiliations:** 1 Department of Molecular Medicine, Scripps Research, 10550 North Torrey Pines Road, La Jolla, CA 92037 USA; 2 Department of Neuroscience, Scripps Research, 10550 North Torrey Pines Road, La Jolla, CA 92037 USA; 3 Sanford Burnham Prebys Medical Discovery Institute, 10901 North Torrey Pines Road, La Jolla, CA 02037 USA

## Abstract

Mutations in the *PARK2* gene are associated with early onset Parkinsonism. The *Park2*^*−/−*^ mouse, however, does not exhibit neurodegeneration or other Parkinson’s disease (PD) phenotypes. Previously, we discovered that translation of Mcl-1, a pro-survival factor, is upregulated in the *Park2*^*−/−*^ mouse, suggesting a compensatory mechanism during development. Here we generated the *Park2*^*−/−*^ *Mcl-1*^*+/−*^ mouse and show that by reducing *Mcl-1* gene dosage by 50%, the *Park2*^*−/−*^ genotype is sensitized, conferring both dopaminergic neuron loss and motor impairments. We propose that this murine model could be a useful tool for dissecting PD etiology and developing treatment strategies against this neurodegenerative disease.

## Abstract (web summary)

Susanna Ekholm-Reed et al. develop a Parkinson mouse model by reducing *Mcl-1* gene dosage by half, which leads to dopaminergic cell loss and motor impairments in *Park2* knockout mice that otherwise do not exhibit any of the hallmark phenotypes associated with Parkinson’s disease.

## Introduction

Although a number of functions have been attributed to the ubiquitin ligase parkin encoded by the gene *Park2*^1^, we have previously shown that its neuroprotective effect is mediated, at least in part, by targeting the substrate binding adaptor of another ubiquitin ligase, SCF^Fbw7β^, for ubiquitin-mediated proteasomal degradation^2^. The critical target of the SCF^Fbw7β^ ubiquitin ligase, in this context, was shown to be the Bcl-2 family member Mcl-1, essential for neuronal survival^3^. During stress, parkin mediated degradation of Fbw7β is critical for maintaining Mcl-1 levels, thereby allowing neurons to survive^2^. Loss of parkin prevents this stress-induced reduction of Fbw7β levels and renders neurons susceptible to apoptosis^2^. However, we found that this only occurred in mouse neurons when *Park2* was acutely silenced. Paradoxically, neurons from *Park2*^*−*/*−*^ embryos were no more sensitive to stress than wild-type neurons and even had slightly elevated basal levels of Mcl-1 even though the rate of Mcl-1 turnover was greater than in wild-type neurons^2^. It has been shown that translation of Mcl-1 is regulated by the mTORC1 complex via phosphorylation of translation initiation inhibitor 4EBP1^4^. Indeed, neurons from *Park2*^*−*/*−*^ mice exhibit hyperactivation of the mTORC1 complex^2^. We therefore hypothesized that *Park2*^*−*/*−*^ embryos may compensate for excessive turnover of Mcl-1 by increasing Mcl-1 translation, explaining why *Park2*^*−*/*−*^ mice fail to exhibit a neurodegenerative phenotype.

In this paper, we show that hemizygous deletion of *Mcl-1* in the mouse sensitizes the *Park2* null genotype, producing a phenotype that resembles human PD. This genetic strategy overcomes an intrinsic compensatory mechanism that increases Mcl-1 translation in the absence of parkin, shielding mice from most of the deleterious effects of parkin loss. *Park2*^*−*/*−*^ *Mcl-1*^*+/−*^ mice exhibit progressive motor function deficits and neurodegeneration specifically in the substantia nigra, as is the case with humans suffering from PD. This new model should be useful in exploring the etiology of PD as well for developing therapeutics to treat the disease.

## Results

### Increased Translation of Mcl-1 in neurons from Park2^−/−^ mice

**Fig. 1:**
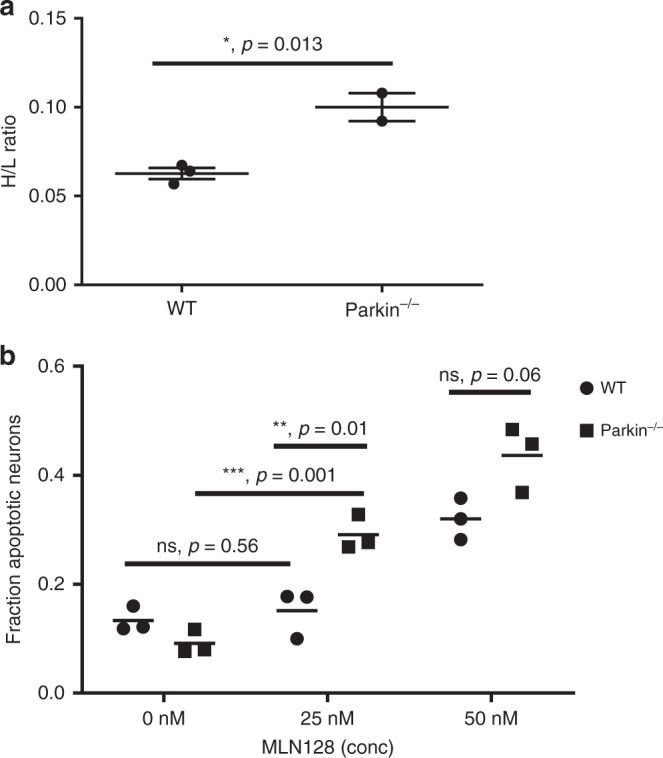
Mcl-1 translation is enhanced and apoptosis reduced in *Park2*^−/−^ neurons due to mTOR activation. **a** *Park2*^−/−^ and wild-type primary, embryonic mouse neurons were pulse labelled with 13 C, 15N lysine and arginine, lysates were immunoprecipitated using anti-Mcl-1 antibody and tryptic peptides analyzed for heavy to light ratio. The results are the composite of two biological experiments. **b** *Park2*^*−/−*^ and WT primary, embryonic mouse neurons were treated with the mTOR inhibitor MLN128 and the oxidant NOC12 for 24 h. The percentage of apoptotic cells was determined based on DAPI staining and nuclear morphology. All error bars correspond to SEM. Significance determined by two-tailed *t* test

**Fig. 2:**
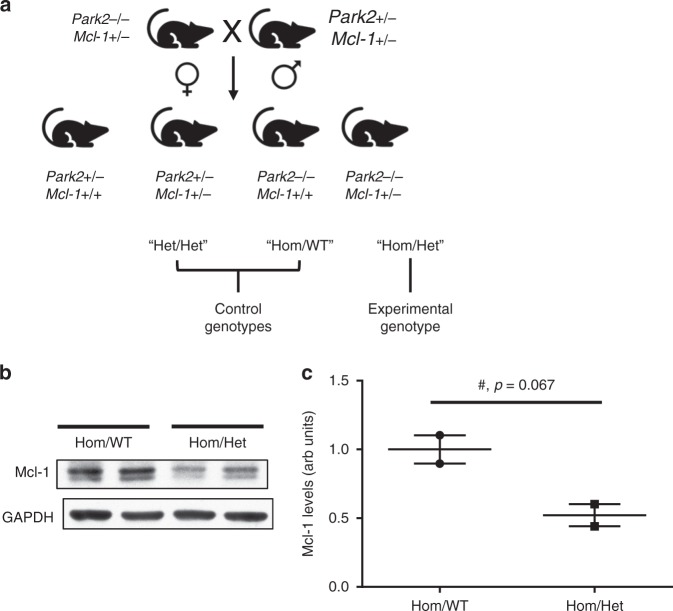
Generation of the *Park2*^−/−^ *Mcl-1*^*+/−*^ mouse. **a** Breeding scheme. **b** Western blot analysis of Mcl-1 expression in brain lysates. GAPDH, loading control. **c** Quantification of the western blot in (b). Error bars correspond to SEM. Significance determined by two-tailed t test

We determined the relative rate of Mcl-1 translation in *Park2*^*−*/*−*^ versus wild-type neurons by pulsed SILAC and mass spectrometry. As we had already shown that Mcl-1 steady state levels are not reduced in Park2^*−*/*−*^ neurons^2^, we could infer from the ratio of heavy to light amino acids incorporated that the rate of Mcl-1 synthesis was significantly increased in *Park2*^*−*/*−*^ compared to wild-type neurons (Fig. 1a). Conversely, when calculating the H/L ratios of specific individual proteins and averaging the H/L ratios of 52 proteins that were non-specifically precipitated with the Mcl-1 immune complexes, no differences were observed when comparing *Park2*^*−*/*−*^ and wild-type neurons (Supplementary Fig. 1). Mcl-1 translation has been shown to be positively regulated by mTORC1 activation^4^ and we have shown that mTORC1 is activated in *Park2*^*−*/*−*^ neurons^2^. These observations are consistent with a germline mTORC1-dependent compensatory mechanism to maintain Mcl-1 levels in *Park2*^*−*/*−*^ neurons, possibly accounting for the lack of a neurodegenerative phenotype in *Park2*^*−*/*−*^ mice. In support of this hypothesis, when cultures of wild-type and *Park2*^*−*/*−*^ neurons were treated with low doses of the mTOR inhibitor MLN128 (25 nM)^5^, *Park2*^*−*/*−*^ neurons exhibited increased sensitivity to oxidative stress, whereas wild-type neurons were not significantly affected (Fig. 1b). This correlated with a much greater decrease in Mcl-1 levels in *Park2*^*−*/*−*^ neurons than in wild-type neurons (Supplementary Fig. 2). At higher doses, both *Park2*^*−*/*−*^ and wild-type neurons were affected, as would be expected. These results suggest that a neurodegenerative phenotype might be achieved in the *Park2*^*−*/*−*^ mouse by impairing the ability of neurons to compensate. To test this hypothesis, we created the *Park2*^*−*/*−*^ *Mcl-1*^*+/−*^ (Hom/Het) mouse (Fig. 2a). Germline deletion of *Mcl-1* has been shown to be embryonic lethal when homozygous but aphenotypic when heterozygous^6^. *Park2*^*−*/*−*^ *Mcl-1*^*+/+*^ (Hom/WT) and *Park2*^*+/−*^ *Mcl-1* + */−* (Het/Het) were bred as controls (Fig. 2a). Western analysis confirmed a 50% reduction of Mcl-1 protein levels in Hom/Het brains (Fig. 2b, c).

### Motor impairment in the *Park2*^*−/−*^*Mcl-1*^*+/−*^ mouse

**Fig. 3:**
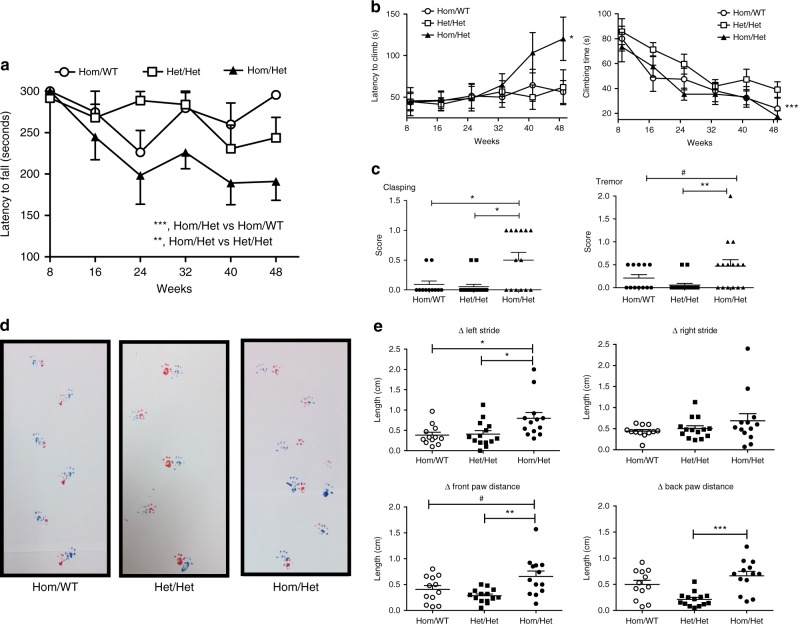
The *Park2*^−/−^, *Mcl-1*^*+/−*^ mouse exhibits PD phenotypes. **a** Rotarod performance over a 48-week time course. Data as mean + /- S.E.M. performance for male mice only. Two-way ANOVA revealed significant differences between Hom/Het mice compared to both Hom/WT mice (***p = 0.0008; F(1,75) = 12.33) and Het/Het mice(**p = 0.002; F(1,85) = 10.69. **b** Differences in climbing behavior (climbing latency and climbing time) of the indicated genotypes (both males and females). Two-way ANOVA revealed significant differences between Hom/Het mice compared to Hom/WT mice (*p = 0.040; F(1,124) = 4.2) and Het/Het (*p = 0.037; F(1,132) = 4.4) for climbing latency. A significant difference in climbing time between Hom/Het mice and Het/Het mice (***p = 0.0008; F(1,132) = 11.8) was also observed. **c** Hom/Het mice show significant differences in clasping and tremor compared to both control genotypes. #, P = 0.07; *P = 0.01; **P = 0.009, as determined by two-tailed Student’s *t* test. **d**, **e** Mice were tested for footprint analysis at 52 weeks of age. **d** Representative tracing from each of the three genotypes. **e** Scatter graph quantification of the differences in left and right stride length (top graphs) and front and rear paw distances (bottom graphs). *P < 0.05; **P < 0.005; ***P < 0.0001, as determined by two-tailed Student’s *t* test. Left stride difference Hom/Het versus Het/Het, *p* = 0.022; Hom/Het versus Hom/WT, *p* = 0.0173. Back paw difference Hom/Het versus Het/Het, *p* = 0.00009. Front paw difference Hom/Het versus Het/Het, *p* = 0.0017; Hom/Het vs. Hom/WT, *p* = 0.064

Hom/Het animals were viable, fertile, exhibited normal litter sizes, and weighed the same as control littermates at the endpoint of the experiment (52 wks; Supplementary Fig. 3). Motor function and behavioral tests were performed every 8 weeks. Starting at 16 weeks of age, a significant reduction in latency to fall on the rotarod test was observed in Hom/Het mice, as compared to control mice (Fig. 3a; Supplementary Data 1). Hom/Het mice also exhibited reduced activity in the open field when compared to Het/Het and/or Hom/WT mice. These effects were manifested largely as decreased vertical time, vertical counts, jumping time and jumping counts (Supplementary Table 1). Climbing behavior, assessed by measuring latency to climb and climbing time, were significantly increased and decreased, respectively, in the Hom/Het animals, compared to control groups (Fig. 3b; Supplementary Data 2). At 52 weeks of age, Hom/Het mice exhibited abnormal hindlimb clasping (associated with motor impairment in other neurodegenerative mouse models) and tremor (Fig. 3c). Gait abnormalities were assessed by analyzing footprint patterns, indicating that the Hom/Het mice are significantly impaired (Fig. 3d, e). Notably, these abnormalities are reminiscent of gait deficiencies in PD patients^7^.

### Park2^−/−^ Mcl-1^+/−^ mice exhibit neurodegeneration

**Fig. 4:**
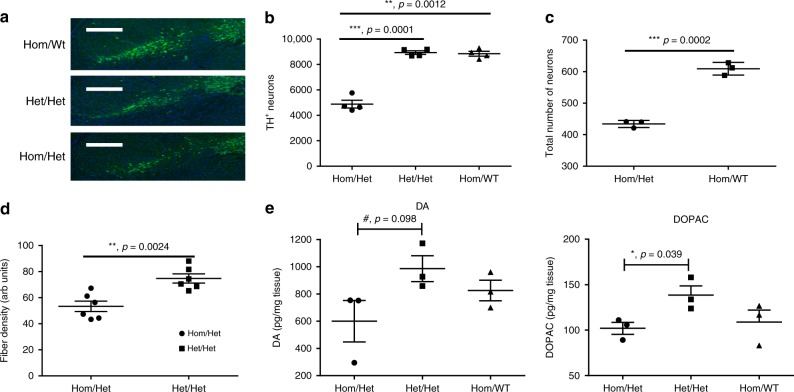
The *Park2*^−/−^, *Mcl-1*^*+/−*^ mouse exhibit dopaminergic neurodegeneration. **a** TH^+^ immunofluorescence staining of equivalent SN sections from Hom/WT, Het/Het and Hom/Het brains. Scale bars, 200 µm. **b** The estimated total number of TH + neurons in equivalent sections of the SN, in 52-week old mice, *n* = 3. **c** The estimated total number of neurons based on Nissl staining of select equivalent sections of the SN, in 52-week-old mice, *n* = 3. **d** Optical density of TH^+^ fibers in equivalent sections of the striatum of 52-week-old mice. **e** Striatal content of DA and DOPAC in 52-week-old mice. (a–e) Error bars correspond to SEM. *p < 0.05; **p < 0.005; ***p < 0.0001, as determined by two-tailed *t* test

Although these behavioral and motor deficits are consistent with Parkinsonism, they may not be attributable to degeneration of dopaminergic neurons. Therefore, the number of dopaminergic neurons in the substantia nigra (SN) was assessed by tyrosine hydroxylase (TH) immunofluorescence and direct counting at 52 weeks. The number and density of TH^+^ neurons within the SN was significantly reduced in Hom/Het mice relative to the controls (approximately 45%) (Fig. 4a, b). The control numbers (approximately 9000 TH^+^ neurons per SN) are similar to literature values for wild-type mice^8^. Total neuron counts based on Nissl staining confirmed that loss of TH staining observed in Hom/Het mice is due to neuronal death and not loss of TH expression (Fig. 4c). Furthermore, we observed a reduction of TH^+^ fibers projecting into the striatum, consistent loss of dopaminergic neurons in the SN (Fig. 4d). Finally, also consistent with loss of dopaminergic neurons, dopamine and a dopamine metabolite, 3,4-dihydroxyphenylacetic acid (DOPAC), were both found to be reduced in Hom/Het striata, compared to in controls (Fig. 4e).

## Discussion

The data presented suggest that hemizygous deletion of *Mcl-1*, eliminating a compensatory pathway in the mouse, may potentiate a *Park2*^*−/−*^ phenotype that truly mirrors inherited recessive PD. We have shown that reducing the gene dosage of Mcl-1 by 50% in the *Park2*^*−/−*^ mouse produces neurodegenerative and PD-like phenotypes, similar to human PD. This result provides *in vivo* support for the idea that Parkin promotes neuronal survival in part by targeting Fbw7β, thereby stabilizing Mcl-1. Parkin has also been shown to regulate the clearance of damaged mitochondria, known as mitophagy^9–11^. Consistent with this, a recently described *in vivo* model that combines *Park2* deletion with a mutator allele of the mitochondrial DNA polymerase, POLG, exhibits neurodegeneration in the SN and motor deficits at about a year of age^12^, similar to what we have reported here. This result is consistent with the idea that the inability to clear non-functional mitochondria resulting from accumulated mtDNA mutations, is stressful to nigral neurons leading to their death. More recently it has been shown that neuron loss in that model is due to the cytosolic exposure of double stranded, presumably mitochondrial, DNA and concomitant triggering of the innate immune response and inflammation^13^ in the absence of efficient mitophagy^14^. It will be interesting to determine whether nigral neuron loss in the current model is due to inflammation or to other chronic stresses that are lethal in the absence of optimal Mcl-1 levels.

The creation of a non-targeted rodent PD model using biallelic *Park2* mutation is important in that it provides a useful PD-related *in vivo* model for investigating how germline parkin deficiency leads to neurodegeneration. Furthermore, since the neuropathology of PD due to biallelic *PARK2* mutation and that of idiopathic PD in humans is similar, insights gained are likely to be useful for understanding PD in general. The *Park2*^*−/−*^*Mcl-1*^*+/−*^ mouse is also important in that it will provide a useful tool for PD drug discovery.

## Methods

### Generation of *Park2/Mcl-1* mice

Mice of genotype *Mcl1*^*+/−*^^6,15^ (a beta-GEO insertion replacing most of exons 1 and 2, a gift of Joseph Opferman, St. Jude Children’s Research Hospital) were crossed with *Park2*^*−/−*^ (deleted for exon 3, ref) mice. Both mouse strains were on a C57BL/6 background. Double heterozygotes were then intercrossed to generate the *Park2*^*−/−*^ *Mcl1*^*+/−*^ (Hom/Het) genotype, as well as *Park2*^*−/−*^, *Mcl-1*^*+/+*^ (Hom/WT) and *Park2*^*+/−*^ *Mcl-1*^*+/−*^ (Het/Het) controls. Colonies of these mice have been maintained at The Scripps Research Institute. Mouse work was compliant with NIH and Scripps Research Institute guidelines (The Scripps Research Institute is AAALAC accredited) and was approved by the Scripps Research Institute Animal Research Committee.

### Pulsed SILAC and analysis of Mcl-1 peptides

Labeling and immunoprecipitation: DIV 6 primary mouse embryonic brain neurons from wild-type or *Park2*^*−/−*^ mice were pulse labeled for 10 min with a mixture of 13 C and 15 N lysine and arginine. Lysates were prepared as described^2^ and immunoprecipitated with anti-Mcl1 rabbit monoclonal antibody (5 μl, D2W9E, Cell Signaling Technology) and Gammabind protein G Sepharose (GE Healthcare).

Sample preparation: Proteins were eluted and digested in 8 M urea 50 mM ammonium bicarbonate buffer. Briefly, cysteine disulfide bonds were reduced with 5 mM tris (2-carboxyethyl)phosphine (TCEP) at 30 °C for 60 min followed by cysteine alkylation with 15 mM iodoacetamide (IAA) in the dark at room temperature for 30 min. Following alkylation, urea was diluted to 1 M urea using 50 mM ammonium bicarbonate, and proteins were subjected to overnight digestion with mass spec grade Trypsin/Lys-C mix (Promega, Madison, WI). Digested proteins were desalted using a C_18_ TopTip (PolyLC, Columbia, MD) according to the manufacturer’s recommendation), and the organic solvent was removed in a SpeedVac concentrator prior to LC-MS/MS analysis.

LC-MS/MS analysis: Dried samples were reconstituted with 2% acetonitrile, 0.1% formic acid and analyzed by LC-MS/MS using a Michrom HPLC system (Michrom-Bruker) coupled to a Orbitrap Velos Pro mass spectrometer (Thermo Fisher Scientific). Peptides were separated using an analytical C_18_ Michrom Magic column 0.075 × 150 mm (Michrom-Bruker) in a 90-min linear gradient of 2–28% solvent B. The mass spectrometer was operated in positive data-dependent acquisition mode. MS1 spectra were measured with a resolution of 60,000, an AGC target of 1e6 and a mass range from 350 to 1400 m/z. Up to 5 MS2 spectra per duty cycle were triggered, fragmented by CID, with an AGC target of 1e4, an isolation window of 2.0 m/z and a normalized collision energy of 35. Dynamic exclusion was enabled with duration of 20 s.

Data analysis: All mass spectra from were analyzed with MaxQuant software. MS/MS spectra were searched against the *Mus musculus* Uniprot protein sequence database (version January 2014) and GPM cRAP sequences (commonly known protein contaminants). SILAC mode was enabled to detect heavy and light peptides. Precursor mass tolerance was set to 20 p.p.m. and 4.5 p.p.m. for the first search where initial mass recalibration was completed and for the main search, respectively. Product ions were searched with a mass tolerance 0.5 Da. The maximum precursor ion charge state used for searching was 7. Carbamidomethylation of cysteines was searched as a fixed modification, while oxidation of methionines and acetylation of protein N-termini were searched as variable modifications. Enzyme was set to trypsin in a specific mode and a maximum of two missed cleavages was allowed for searching. The target-decoy-based false discovery rate (FDR) filter for spectrum and protein identification was set to 1%, as described previously^16^.

### Brain extracts

Brian tissue (0.05 g) from frozen adult *Park2*^*−/−*^ and WT mouse brains were homogenized in 500 µl of buffer (50 mM Tris-HCl [pH 7.6], 150 mM NaCl, 1% Triton-X, protease inhibitor cocktail tablet [complete Mini, EDTA-free; Roche Diagnostics]), using Lysing Matrix D (BIO 101 Systems) and a FastPrep FP120 (BIO 101 Systems), for 40 s (setting 6) three times. Homogenates were then centrifuged at 10,000 × *g* for 10 min at 4 °C, and the supernatant was collected, mixed with 4x sample buffer (40% glycerol, 240 mM Tris-HCl, [pH 6.8], 8% SDS, 0.04% bromophenol blue and 5% β-mercaptoethanol), boiled for 3 min and analyzed by western blot analysis.

### Western blot analysis

Proteins were separated by SDS-PAGE electrophoresis and transferred to nitrocellulose membrane using an iBlot (Invitrogen). The membrane was blocked for 1 h with 5% nonfat dry milk in TBS containing 0.1% Tween (TBST) and then probed with primary antibody, Mcl-1 (Cell Signaling D35A5) at 1:1000 and GAPDH (Prosci 3781) at 1:2000 or β-actin (Prosci 3779) at 1:2000 overnight at 4 °C. Blot was washed in TBST, incubated in secondary HRP-conjugated goat anti-rabbit IgG antibody (Jackson Laboratories 111-035-003) at 1:5000 for 1 h at RT and the developed using chemiluminescence reagent (Super Signal West Pico PLUS, Chemiluminescence substrate, Thermo Scientific). Quantification of scanned western blots was done using Image J. Images of original films are shown in Supplementary Figures 4 and 5.

### mTOR inhibition and apoptosis assay

Primary neuronal cultures from E16 *Park2*^*−/−*^ and wild-type mice were prepared as described earlier^2^. Mixed populations of primary neurons were isolated from E16 mouse embryos and plated in high-glucose Dulbecco’s modified Eagle’s medium (DMEM-Glutamax), supplemented with 10% fetal bovine serum (Atlanta Biologicals) and 100 units/ml penicillin and 100 µg/ml streptomycin (Invitrogen). Plastic dishes or glass slides (hemacytometer cover glass; Orbeco, Sarasota, FL, USA), coated with poly-L-lysine (Sigma) and mouse laminin (Invitrogen) or in 6-well culture dishes coated in the same manner were used. The day after preparation, the medium was changed to Neurobasal supplemented with B27 (Invitrogen), 1:400 Glutamax (Invitrogen), and 100 units/ml penicillin and 100 µg/ml streptomycin. Neurons were grown at 37 °C in 3% oxygen and 5%CO2. On the seventh day of *in vitro* culture, cells were treated with MLN128 (a gift from Kevan Shokat, UCSF) and the NO radical donor NOC-12 for 24 h and then fixed in 4% paraformaldehyde for 20 min at RT, washed in PBS and incubated for 10 min with DAPI (50 µg/ml, in PBS). The fraction of apoptotic neurons was determined by direct counting based on apoptotic nuclear morphology, using a Zeiss Axioskop 2 fluorescence microscope. Alternatively, neurons were harvested by trypsinization (0.125%), collected by centrifugation and washed two times in phosphate-buffered saline (PBS). Neuron pellets were stored at −80 °C until analyzed. For lysate preparation, neuron pellets were resuspended in lysis buffer (20mMTris-HCl, pH7.5, 150 mM NaCl, 2 mM EGTA, 1% NP-40, protease inhibitor cocktail [Complete Mini, EDTA-free; Roche Diagnostics]) and incubated on ice for 17 min and disrupted using a sonic probe for 10 s. Soluble extracts were obtained by centrifugation using an Eppendorf centrifuge 5415C at the highest speed for 10 min at 4 °C and retaining the supernatant.

### Motor behavioral assessments

Mice were tested in behavioral tasks described below, until 52 weeks of age, when the mice were sacrificed by intracardial perfusion and brains sectioned and stained for immunohistochemistry determinations. All procedures were in strict accordance with the National Institutes of Health *Guidelines for the Care and Use of Laboratory Animals*. Groups of mice (*n* = 13–18 per genotype) were tested in the following behavioral paradigms at the indicated timepoints.

Rotarod test: Animals were tested on an AccuRotor rotarod (AccuScan Instruments) during the dark phase of the 12 h light–dark cycle using a constant rotation paradigm (12 rpm over 10 min). The time of fall was recorded by computer. Mice were trained on the rotarod at 8 weeks of age, in order to establish a behavioral baseline. Mice were then tested in a single set of three trials every 8 weeks from 16–48 weeks of age.

Open field exploration: Open field exploration was measured in a square plexiglass chamber (27.3 × 27.3 cm) (Med Associates INC). The test chamber is divided into 16 squares (12 outer and 4 inner) of equal areas and includes three 16 photobeam I/R arrays to automatically record movement. Several behavioral parameters (ambulatory time, ambulatory distance, jumping, vertical activity and time spent grooming) were recorded during a 10 min observation period. Mice were tested in the open field test every 8 weeks from 17–49 weeks of age (Supplementary data 2 and Supplementary Table 1).

Climbing test: To assess climbing activity, mice were placed on the floor of a wire cylinder (3¾″ height × 4″ diameter) for 5 min and their behavior monitored as described previously^16^. Briefly, climbing was recorded when two or four paws of the mouse were off the floor of the testing bench. Total climbing episodes, climbing time and climbing latency were recorded. Mice were tested every 8 weeks until 48 weeks of age.

Hindlimb clasping: Each mouse was suspended by its tail for 30 s 10 cm above the ground and monitored for the following: 0, normal hindlimb movements; hindlimbs are fully spread and moving about; 1, intermittent clasping of one hindlimb; 2, intermittent clasping of both hindlimbs; 3, both hindlimbs are fully drawn up to the abdomen. Assessments were made by two independent observers who were blind to genotype. Clasping was assessed at 52 weeks of age.

Tremor: Tremor was scored visually by using the following scale: 0, no tremor; 1, tremor when head is extending; 2, constant head and body tremors. Assessments were made by two independent observers who were blind to genotype. Tremor was scored at 52 weeks of age.

Footprint test: The footprint test was used to compare gait at 52 weeks. Hind- and forefeet of the mice were coated with red and blue nontoxic paints, respectively. The animals were then allowed to walk along a runway (50-cm-long, 10-cm-wide, 10-cm-high) into an enclosed box. The footprint patterns were analyzed for various parameters (measured in centimeters). As described previously, left and right stride length was measured as the average distance of forward movement between each stride^17^. Hind-base and front-base width were measured as the average distance between left and right hind- and front- footprints, respectively^17^. Values were determined by measuring the perpendicular distance of a given step to a line connecting its opposite preceding and proceeding steps. For each step parameter, three values were measured at least 4 consecutive strides, excluding footprints made at the beginning and end of the run.

### Statistical analyses

The rotarod, open field and climbing data were analyzed with a Two-way analysis of variance (ANOVA) using GraphPad Prism Software (San Diego, CA). The footprint test, clasping test and tremor scores were analyzed using One-way ANOVA. Significance was accepted at the 95% probability level. The effect size to detect a 30% decrease in motor activity is based on our previous studies^17,18^. Power calculations show that groups of 9 mice are sufficient to have a 95% chance of detecting a 30% decreases in behavioral symptoms, where *n* = log(0.05)/log(0.7) = 8.4. In all cases where Student’s t test was used, the 2-tailed method was employed.

### Tyrosine Hydroxylase Immunohistochemistry

Mice were sacrificed by cardiac perfusion with 4% paraformaldehyde in a 0.1 m sodium phosphate buffer, pH 7.4. The brains were isolated and further post-fixed in 4% paraformaldehyde at 4 °C for 1 day, followed by cryoprotection in 30% sucrose for 1 days. Brains were stored frozen at −80 °C until sectioning. Coronal sections, 40-μm-thick, starting from Bregma −2.54 mm to −4.16 mm, were cut on a crytostat (Leica CM3050S) and every other section (24 sections in total) was processed for tyrosine hydroxylase (TH) immunohistochemistry. Free-floating sections were pretreated in 0.3% H_2_O_2_ in tris-buffered saline Tris-buffered saline (TBS) for 15 min and in blocking solution (4% bovine serum albumin (BSA) in TBS containing 0.1% Triton X-100) for 2 h at room temperature. Sections were washed three times for 15 min in TBS with 0.1% Triton X-100, followed by incubation overnight at 4 °C with polyclonal rabbit anti-TH (1:500; Abcam ab112) in 4% BSA in TBS containing 0.1% Triton X-100. Sections were then washed with TBS containing 0.1% Triton X-100 and incubated with anti-rabbit secondary antibody (1:500; Alexa Fluor 488) in 4% BSA in TBS containing 0.1% Triton X-100 for 2 h at room temperature in the dark. Sections were then incubated in DAPI for 10 min, washed again and mounted on superfrost plus slides with anti-fade medium (ProLong Gold, Invitrogen).

### Neuron Counting

To quantify TH^+^ neurons, representative images of the whole SN area were captured using a tiling function with a 10x objective on a Keyence BZX700 fluorescence microscope. TH^+^ neurons in the right hemisphere were counted manually, using the counting tool in Photoshop. To quantify the total number of neurons, SN sections were stained with cresyl violet acetate solution (Nissl staining)^19^ and representative images of the right hemisphere SN area were captured on a Keyence BZX700 microscope, using a 20x objective. Images were opened in Photoshop, magnified 200% and neurons within the generated window were counted using the Photoshop counting tool. Only cells with neuronal morphology were counted, for three equivalent sections per brain^20^.

### TH fiber density in the striatum

The fiber density in the striatum was quantified by measuring the mean intensity of TH staining in five corresponding striatal sections for each genotype, using ImageJ software. The staining protocol was the same as for midbrain TH staining described above. The measured values were corrected for non-specific background staining by subtracting values obtained from staining with secondary antibody alone.

### Dopamine Analysis

The striata and cerebellum were homogenized in ice-cold 0.1 mol/L perchloric acid (10 µL per mg tissue) containing 1.34 mmol/L EDTA and 0.05%, w/v sodium bisulfite and sonicated for 2 × 15 s, then the homogenates were centrifuged at 16,000 × *g* for 20 min at + 4 °C, and the supernatants analyzed for monoamine content on the same day. The supernatants were assayed by HPLC and electrochemical detection, using procedures similar to those described previously^19,21^. The HPLC system used was a Hitachi Labchrom 7200 series chromatograph with a room-thermostatted electrochemical Decade Intro detector using a 3 mm glass carbon working electrode, an Ag/AgCl reference electrode, and a 25 µm spacer (cell volume 180 nL, Antec, Leyden, The Netherlands). Runs were performed at room temperature using a 25 cm × 4.6 mm Beckman Coulter Ultrasphere 5 µm C18 column equipped with a C18-filter security guard system (Phenomenex). The mobile phase solution, pumped at a flow rate of 0.8 mL/min, was 0.07 mol/L potassium phosphate, 0.1 mmol/L EDTA, 1.1 mmol/L OSA, 3.1 mmol/L TEA, 14% methanol, pH adjusted to 3.12 with 1 mmol/L citric acid and was filtered using a 0.22 µm cellulose acetate membrane before use. Analytes were detected at an oxidation potential of 700 mV against the reference electrode. Chromatograms were acquired using Hitachi LabChrom software with the standard options of acquisition (10 Hz as the initial rate, then halving the rate every 10 min), as described previously^19^. The acquisition time was 35 min. Samples were placed in the autosampler and kept at + 10 °C prior to injection. The injection volume was 50 µL. The calibration standards for the quantification validation contained NE, DA, DOPAC, HVA, 5-HT, and 5-HIAA (range: 10−9–10−6 mol/L).

### Reporting Summary

Further information on experimental design is available in the Nature Research Reporting Summary linked to this article.

## Supplementary information

Supplementary Information

Supplementary Data 1

Supplementary Data 2

Description of Additional Supplementary Files

Reporting Summary

## Data Availability

The data sets generated during and/or analyzed during the current study not included in Supplementary Data are available from the corresponding author on request. The mouse genotypes described in this study will be made available to the research community upon request.
