# Supplementary material for: Reducing Mcl-1 gene dosage induces dopaminergic neuronal loss and motor impairments in Park2 knockout mice
Source: Commun Biol. 2019 Apr 4;2:125. doi: 10.1038/s42003-019-0366-x (PMC6449387; doi:10.1038/s42003-019-0366-x)
Supplement: Supplementary file 1 — Supplementary Information [file 42003_2019_366_MOESM1_ESM.pdf]

Supplementary Figure 1.

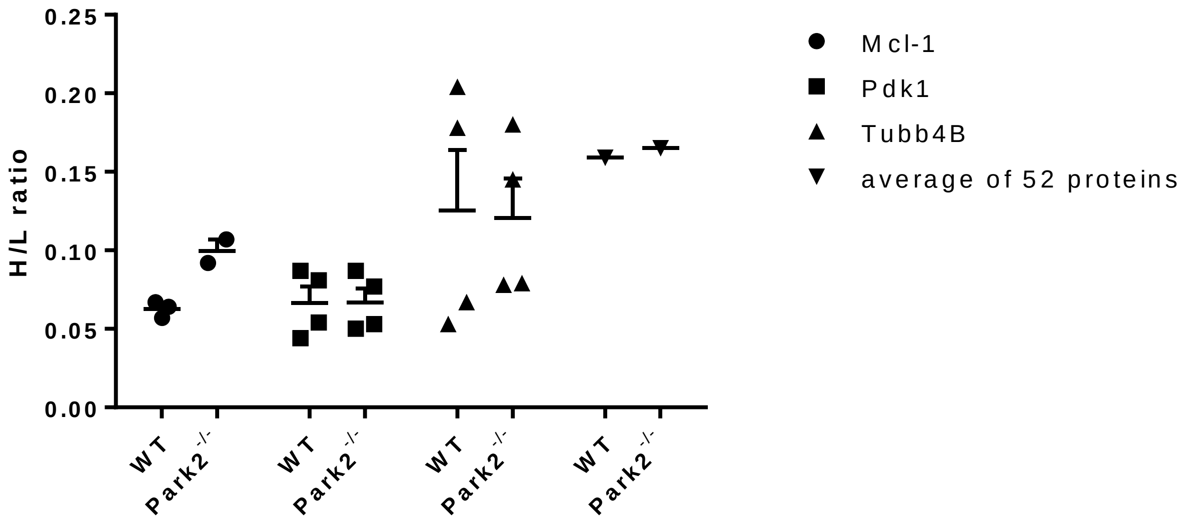

Supplementary Figure 1. ***Parkin*$^{-/-}$ neurons don't exhibit generalized increased translation.** Comparison of heavy/light (H/L) amino acid ratios for Mcl-1 and two other proteins that co-precipitated: Pdk1 (pyruvate dehydrogenase) and Tubb4b (β-tubulin). Also included is the average H/L ratio for 52 proteins.

Supplementary Figure 2.

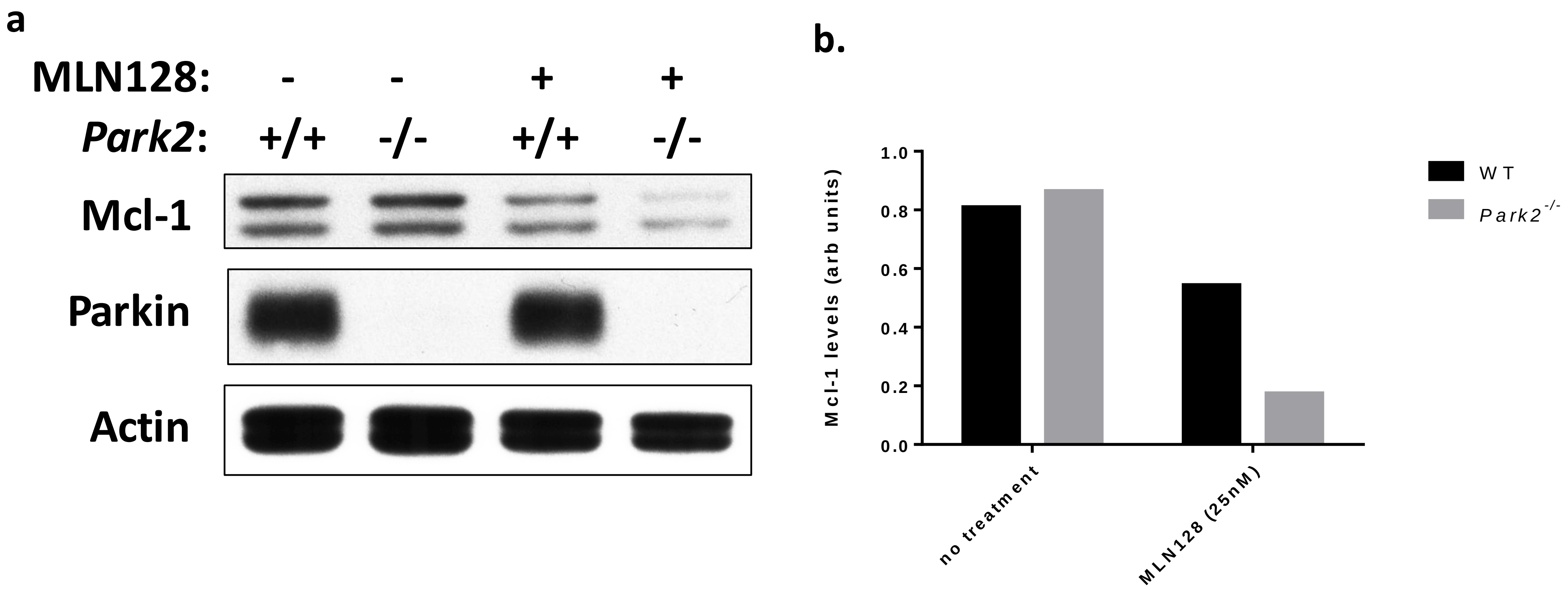

Supplementary Figure 2. Treatment with low concentrations of the mTOR inhibitor MLN128 has a greater impact on Mcl-1 levels in *Park2*$^{-/-}$ neurons than in wild-type neurons. (a) Western blot of neuronal extracts from *Park2*$^{-/-}$ or wild-type primary neurons treated with DMSO or MLN128 (25 nM). **(b)** Quantification of western blot in (a). Graph corresponds to Mcl-1 normalized to actin.

Supplementary Figure 3

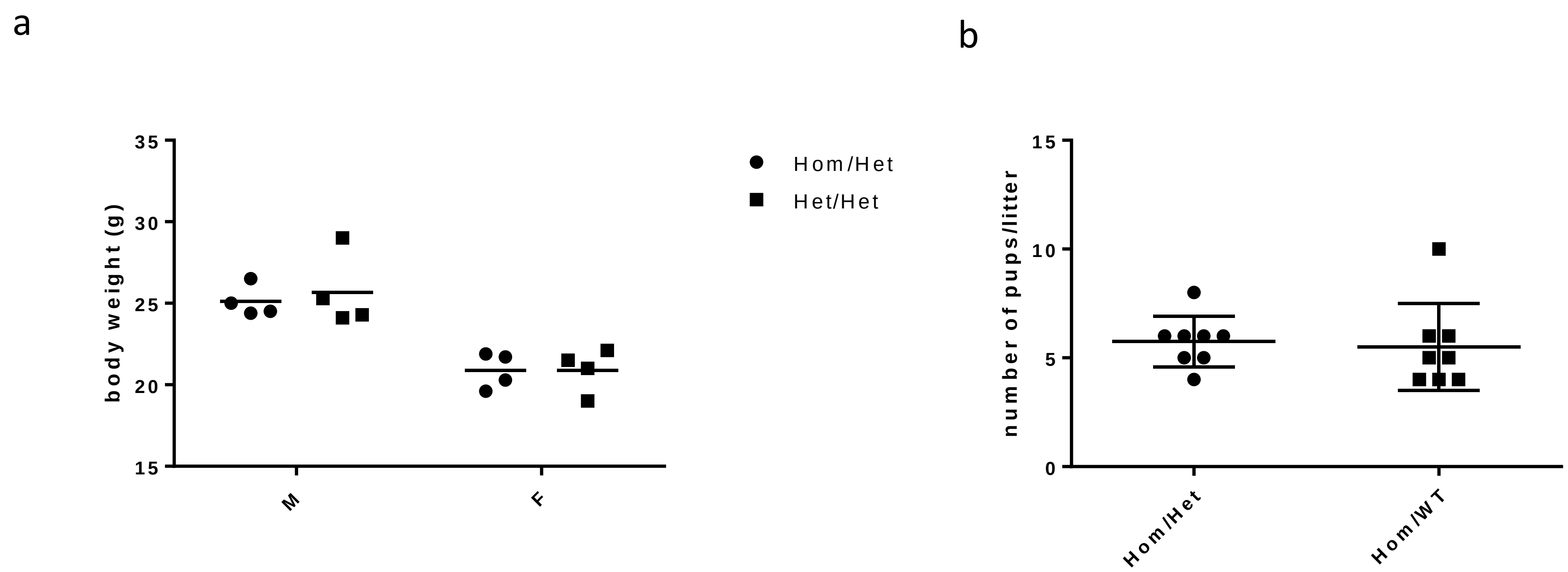

Supplementary Figure 3. Growth and litter size data. (a) Body weights for females (F) and males (M) of Het/Het and Hom/Het genotypes at study endpoint (52 weeks), n=4. Error bars correspond to SD. **(b)** Average litter sizes for Hom/WT and Hom/Het females (two litters per female), n= 4. Error bars correspond to SD**.** Note that Het/Het females are not included as they were not used for breeding.

Supplementary Fig. 4

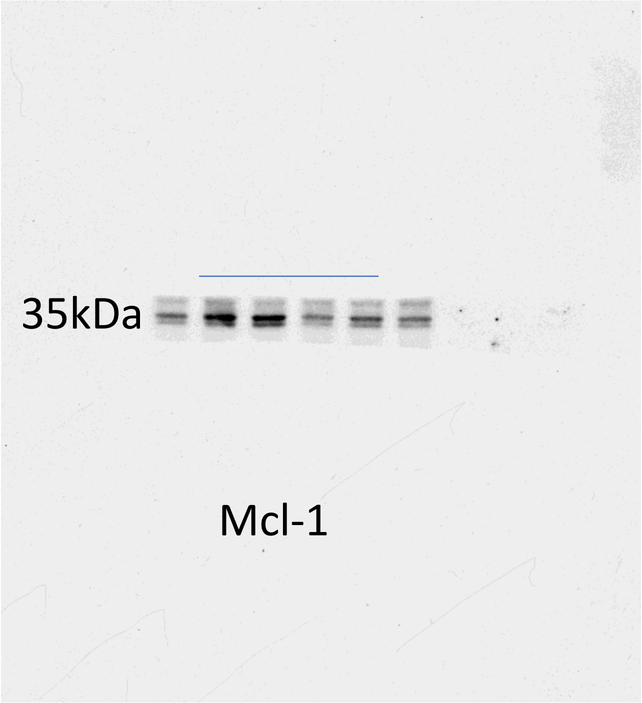

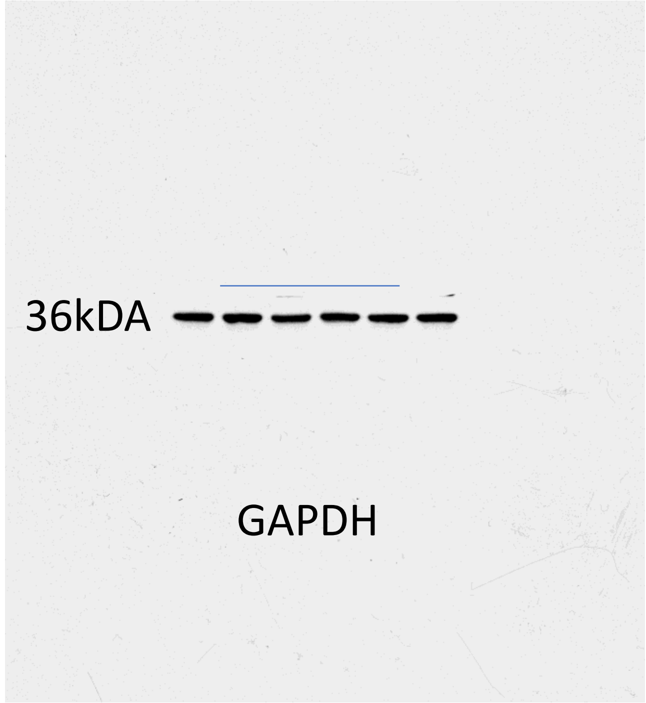

Supplementary Figure 4. Original films for western blot in Figure 2. Bar indicates the lanes used in the figure.

Supplementary Fig. 5

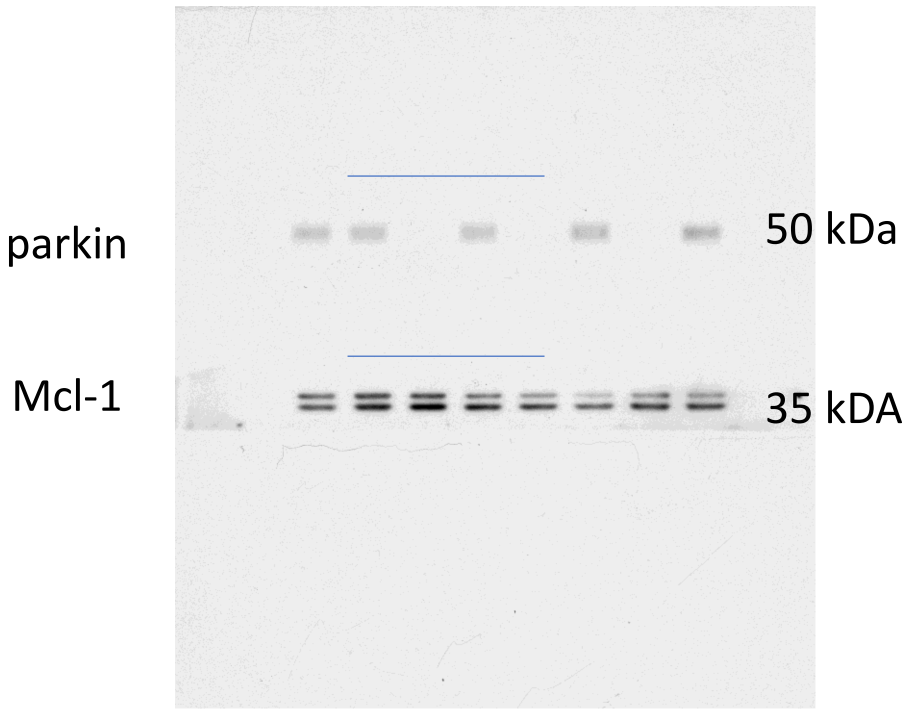

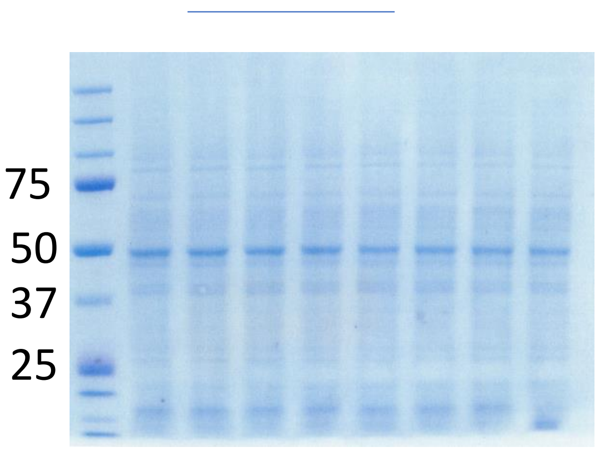

Imido black

Supplementary Figure 5. Original film for western blot in Supplemental Figure 2. Bar indicates the lanes used in the figure. Imido black staining of entire blot is included to confirm evenness of loading.

Supplementary Table 1. Summary of open field data of Park2/Mcl1 mice.

	**Jump Time:**							
	**24 weeks**		**32 weeks**		**40 weeks**		**48 weeks**	
**Comparison:**	**P-value:**	**Effect:**	**P-value:**	**Effect:**	**P-value:**	**Effect:**	**P-value:**	**Effect:**
Hom/Het vs. Het/Het	NS	No change	0.0024	Decrease	0.11	Decrease	0.0001	Decrease
Hom/Het vs. Hom/WT	NS	No change	NS	No change	0.015	Decrease	NS	No change
	**Jump Counts:**							
	**24 weeks**		**32 weeks**		**40 weeks**		**48 weeks**	
**Comparison:**	**P-value:**	**Effect:**	**P-value:**	**Effect:**	**P-value:**	**Effect:**	**P-value:**	**Effect:**
Hom/Het vs. Het/Het	NS	No change	0.0002	Decrease	0.024	Decrease	0.0011	Decrease
Hom/Het vs. Hom/WT	NS	No change	NS	No change	NS	No change	NS	No change
	**Vertical Time:**							
	**24 weeks**		**32 weeks**		**40 weeks**		**48 weeks**	
**Comparison:**	**P-value:**	**Effect:**	**P-value:**	**Effect:**	**P-value:**	**Effect:**	**P-value:**	**Effect:**
Hom/Het vs. Het/Het	0.044	Decrease	0.0028	Decrease	0.041	Decrease	0.0098	Decrease
Hom/Het vs. Hom/WT	0.018	Decrease	0.043	Decrease	NS	No change	NS	No change
	**Vertical Counts:**							
	**24 weeks**		**32 weeks**		**40 weeks**		**48 weeks**	
**Comparison:**	**P-value:**	**Effect:**	**P-value:**	**Effect:**	**P-value:**	**Effect:**	**P-value:**	**Effect:**
Hom/Het vs. Het/Het	NS	No change	0.0001	Decrease	0.003	Decrease	0.0084	Decrease
Hom/Het vs. Hom/WT	0.049	Decrease	0.035	Decrease	NS	No change	NS	No change

Mice were subjected to the open field apparatus for 10 minutes every 8 weeks of age. P-values for the indicated comparisons at 24, 32, 40 and 48 weeks are shown. The "Effect" shows the Hom/Het performance compared to Het/Het and Hom/WT. Statistical differences were determined by Two-way ANOVA.
