# Supplementary material for: Reducing Mcl-1 gene dosage induces dopaminergic neuronal loss and motor impairments in Park2 knockout mice
Source: Commun Biol. 2019 Apr 4;2:125. doi: 10.1038/s42003-019-0366-x (PMC6449387; doi:10.1038/s42003-019-0366-x)
Supplement: Supplementary file 4 — Description of Additional Supplementary Files [file 42003_2019_366_MOESM4_ESM.docx]

**Supplementary Data 1.** Raw data used to generate Figures 3A and B.

**Supplementary Data 2.** Raw data used to generate Supplemental Table 1.
